# An activation-induced IL-15 isoform is a natural antagonist for IL-15 function

**DOI:** 10.1038/srep25822

**Published:** 2016-05-11

**Authors:** Lei Zhao, Bo Hu, Yinsheng Zhang, Yuan Song, Dandan Lin, Yonghao Liu, Yu Mei, Dedy Sandikin, Weiping Sun, Min Zhuang, Haiyan Liu

**Affiliations:** 1 Institute of Blood and Marrow Transplantation, Cyrus Tang Hematology Center, Department of Hematology, Collaborative Innovation Center of Hematology, the First Affiliated Hospital of Soochow University, Suzhou 215006, P. R. China; 2 Immunology Programme, Life Sciences Institute and Department of Microbiology and Immunology, National University of Singapore, Singapore 117456, Singapore; 3 School of Life Science and Technology, ShanghaiTech University, Shanghai 201210, P. R. China

## Abstract

Interleukin 15 (IL-15) expression induces the secretion of inflammatory cytokines, inhibits the apoptosis of activated T cells and prolongs the survival of CD8^+^ memory T cells. Here we identified an IL-15 isoform lacking exon-6, IL-15ΔE6, generated by alternative splicing events of activated immune cells, including macrophages and B cells. *In vitro* study showed that IL-15ΔE6 could antagonize IL-15-mediated T cell proliferation. The receptor binding assay revealed that IL-15ΔE6 could bind to IL-15Rα and interfere with the binding between IL-15 and IL-15Rα. Over-expression of IL-15ΔE6 in the murine EAE model ameliorated the EAE symptoms of the mice. The clinical scores were significantly lower in the mice expressing IL-15ΔE6 than the control mice and the mice expressing IL-15. The inflammation and demyelination of the EAE mice expressing IL-15ΔE6 were less severe than the control group. Furthermore, flow cytometry analysis demonstrated that IL-15ΔE6 expression reduced the percentages of inflammatory T cells in the spleen and spinal cord, and inhibited the infiltration of macrophages to the CNS. Our results demonstrated that IL-15ΔE6 could be induced during immune activation and function as a negative feedback mechanism to dampen IL-15-mediated inflammatory events.

Interleukin 15 (IL-15) is a glycoprotein of the four-α-helix bundle family^1^,^2^,^3^ that is key to immune regulation^4^,^5^,^6^. IL-15 is required for development, homeostatic proliferation and activation of NK and NKT cells as well as intraepithelial lymphocytes (IELs)^7^,^8^,^9^. Furthermore, studies indicate that IL-15 promotes long-term maintenance of CD8^+^ memory T cell proliferation and enhances cytotoxicity of CD8^+^ T cells^10^,^11^,^12^. Moreover, IL-15 can promote the proliferation of neutrophils, mast cells and B lymphocytes and increase phagocytosis of neutrophils, macrophages and dendritic cells and induce their cytokine productions^13^,^14^,^15^,^16^. Thus, IL-15 is a pleiotropic cytokine with many potential regulatory functions during immune responses.

As a potent immunomodulator, the expression of IL-15 is tightly controlled and its mRNA is distributed across various cell types and tissues, including activated monocytes/macrophages, DCs, epithelial cells, and placenta, kidney, lung, heart, and skeletal muscle^17^,^18^. Although IL-15 mRNA expression is widespread, measuring sufficient quantities of IL-15 in the culture supernatant is challenging due to the regulation of IL-15 protein production^18^,^19^. Multiple AUG sequences in the 5′-UTR, regulatory sequence in the C terminus of IL-15 mRNA reduce IL-15 translation^19^,^20^,^21^. Alternative splicing also regulates IL-15 expression and previous work confirms that five alternative spliced forms of IL-15 mRNA have been identified, three of which encode an identical mature IL-15 protein with differences only in signal peptide coding regions^19^,^21^,^22^,^23^,^24^. The other two isoforms were found in mouse intestinal epithelia, lacking exon 6 or a portion of exon 7, and both encode in-frame IL-15 proteins^25^. Although the lacking exon-6 and -7 isoforms are thought to inhibit IL-15-mediated cell proliferation *in vitro*, it is unclear whether they can be produced by immune cells other than intestinal epithelium or whether their expression is physiologically relevant in disease models.

IL-15 is clinically relevant for autoimmune diseases. Serum IL-15 level is increased in the patients with psoriasis, inflammatory bowel disease, systemic lupus erythematosus, inflammatory synovitis, asthma bronchiale, diabetes mellitus, and autoimmune vasculitis^26^,^27^,^28^,^29^,^30^,^31^. Anti-IL-15 antibodies have been investigated to treat rheumatoid arthritis^32^ and antibody blockade of IL-15 reverses the autoimmune intestinal damage in celiac disease model^33^.

Multiple sclerosis (MS), an inflammatory disease of the central nervous system, is thought to arise from autoimmunity and cytokine dysregulation^34^,^35^,^36^,^37^. IL-15 is critical for the pathogenesis of MS^38^,^39^,^40^ as IL-15 up-regulation occurs in the serum and cerebrospinal fluid of MS patients^41^,^42^ and mice with experimental autoimmune encephalomyelitis (EAE)^2^. However, the role of IL-15 in MS and EAE is controversial: IL-15 knockout mice exhibit aggravated symptoms of EAE^43^,^44^. On the other hand, IL-15 produced by astrocytes can induce CD8^+^ T cell activation to contribute to tissue damage during MS pathogenesis^45^. γδT cell-producing IL-15 also induces memory T cells which transform into pathogenic Th17 cells in EAE mice, so IL-15 could be pathogenic in EAE^46^.

In the current study, we found that exon-6-lacking IL-15ΔE6 isoform was induced in the activated immune cells. This natural-occurring isoform of IL-15 could inhibit the IL-15-mediated T cell proliferation in a dose-dependent manner. The receptor binding assay revealed that IL-15ΔE6 could bind to the IL-15Rα and may compete with IL-15 for binding. We then investigated the role of this isoform in the EAE model. Our results showed that over-expression of IL-15ΔE6 isoform in the EAE mice could significantly reduce the severity of the disease. The infiltrations of pathogenic T lymphocytes and macrophages into the CNS were also prevented by the expression of IL-15ΔE6. Taken together, we identified a natural-occurring antagonist of IL-15, IL-15ΔE6, which could be induced by alternative splicing in activated B cells and macrophages. It could be a potential therapeutic agent for treating autoimmune diseases.

## Material and Methods

### Animals

Specific pathogen-free male C57BL/6 mice (aged 6–8 wk) were purchased from Shanghai Laboratory Animal Center (Shanghai, China). Mice were kept in a specific pathogen-free facility in microisolator cages. All experimental methods were in accordance with the National Animal Care and Use Regulation. All animal protocols were approved by the Institutional Laboratory Animal Care and Use Committee at Soochow University.

### Cell lines and reagents

The murine myeloma cell line SP2/0, monocyte/macrophage cell line Raw246.7, CHO cell line and IL-2/IL-15 dependent cell line CTLL-2 were purchased from Chinese Academy of Sciences Shanghai Institute of Cell Resource Center) (Shanghai, China). Cells were maintained in complete RPMI media. Recombinant human (rh) IL-15 was purchased from Xiamen Special Treasure Biological Engineering (Xiamen, China). Cycloheximide was purchased from Beyotime Biotechnology (Shanghai, China). LPS was obtained from Sigma-Aldrich Co. (St. Louis, MO). Polyclonal anti-IL-15 antibody was purchased from ABcam (Cambridge, MA).

### Cloning and protein expression of IL-15 isoforms

mRNA was prepared from the spleen cells using TRIzol reagent (Invitrogen, Carlsbad, CA). First-strand cDNA was synthesized from 2 μg mRNA using reverse transcriptase (Fermentas, Glen Burnie, MD) and oligo (dT) primers. The synthesized first-strand cDNA (2 μl) was amplified by means of the PCR using 20 pmol of each primer specific for murine IL-15 with 2.5 UrTaq (Thermo Fisher Scientific, Waltham, MA) in a total volume of 50 μl reaction buffer consisting of 10 Mm Tris-HCl, 50 mM KCl, and 1.5 mM MgCl_2_. The specific primers used were as follows: IL-15 subcloned into pET43.1 vector: sense, 5′-GAGCTCCCAACTGGATAGATGTAAG-3′, and antisense, 5′-CTCGAGTTA GGACGTGTTGATGAACA-3′. The products were resolved on 1.5% agarose gels and visualized by staining with ethidium bromide. The PCR products were subcloned into pET43.1vector (Invitrogen, Carlsbad, CA). Recombinant plasmids were selected by restriction enzyme digestion and sequenced. The proper recombinant pET43.1 plasmids containing mature protein-coding sequences of IL-15, or IL-15ΔE6 were transformed into BL21 (DE3). Single clone was picked and cultured in complex auto-inducing media at 37 °C for 16 h^47^. The bacteria were harvested and proteins were purified by affinity chromatography through Ni-NTA Agarose (Qiagen, Duesseldorf, Germany).

### CTLL-2 assay

CTLL-2 cells were used 2 days after the last feeding. For the assay, CTLL-2 cells were washed three times in medium without IL-2. Prepare dilutions of purified IL-15, IL-15ΔE6, or commercially bought IL-15 in RPMI1640 complete medium in 96-well plates followed by addition of 5 × 10^4^ CTLL-2 cells/100 μl/well. For the blocking assay, 40 μg/ml purified IL-15, 2.5 ug/ml commercially bought rhIL-15 or 1.25 ug/ml LPS was added into the wells containing titrated purified IL-15ΔE6 or IL-15. In the bioassays for binding activity, the mixture of IL-15Rα/IL-15 or IL-15Rα/IL-15ΔE6 were added in the medium. In a separate experiment, increasing amounts of IL-15ΔE6 were added to the mixture of IL-15Rα/IL-15. The CTLL-2 cells were then added. The plates were incubated in a humidified 37 °C, 5% CO_2_ incubator a total of 24 h. 1 μCi/well [^3^H] thymidine (Shanghai Institute of Physics, Chinese Academy of Sciences, Shanghai, China) was added to the culture for the last 8 h of the assay. Cells were harvested and [^3^H] thymidine was measured on a beta plate reader (PerkinElmer Instruments, Meriden, CT).

### Protein interaction assay

The CheckMate™/Flexi^®^ Vector Mammalian Two-Hybrid System (Promega, Fitchburg, WI) was used to validate interactions between IL-15ΔE6 and IL-15Rα and compare the affinity of IL-15/IL-15Rα with IL-15ΔE6/IL-15Rα. The coding regions of IL-15 or IL-15ΔE6 were transferred into pFN10A (ACT) vector and the coding regions of IL-15Rα was transferred into pFN11A (BIND) vector. These constructs and Pgl4.31[luc2P/GAL4UAS/Hygro] vector were purified and combined and transfected into CHO cell line. The pACT and pBIND vectors were transfected together as negative control. The pACT-MyoD and pBINDId control vectors were used together as positive control. The bioluminescent signal from this mammalian two-hybrid experiment was measured by microplate reader (Winooski, VT). Renilla luciferase activity expressed from the BIND-type vectors can be used to normalize transfection efficiency. The binding ratio was calculated as follows: firefly luciferase reading/Renilla luciferase reading.

### Hydrodynamic gene transfer

The coding region of IL-15 or IL-15ΔE6 was amplified by RT-PCR and inserted into minicircle (MC) plasmid (pMC.EF1, SBI, Mountain View, CA). Plasmid DNA was purified by Maxi-prep Kit (Axygen, Union City, CA). For hydrodynamic gene transfer, the mice were injected i.v. with 25 μg of the recombinant plasmid in a total of 1 ml saline solution within 5s using a 23-gauge needle three days before disease induction.

### Flow cytometry analysis

Single cell suspensions obtained from spleen and CNS were analyzed using flow cytometry. Anti-murine CD16/CD32 FcR block (2.4G2) and all of the following Abs against murine Ags were purchased from BD Biosciences (San Diego, CA): anti-mouse CD11c-FITC, Gr1-PerCP/Cy5.5, CD11b-APC, CD25-APC, CD3-PECF594, CD4-APC/H7, CD8-APC, CD127-PE, NK1.1-PerCP/Cy5.5. All staining were performed in FACS buffer (1 × PBS, 1% BSA, and 0.1% NaN_3_) in the presence of purified anti-CD16/32 at saturation to block unspecific staining for 30 min at 4 °C. Intracellular staining was performed after the cells were incubated with 10 mg/ml brefeldin A for 4 h. Cells were fixed with 4% paraformaldehyde and permeabilized with BD Perm/Wash buffer (BD Biosciences, San Jose, CA) and stained for IFN-γ, TNFα and IL-17.Seven-color flow cytometric analyses were performed using a FACS Canto II flow cytometer (BD Biosciences, San Jose, CA) and analyzed using Flowjo software (Tree Star, Ashland, OR).

### Western blotting

Murine splenocytes lysate or purified proteins were applied to 10–20% SDS polyacrylamide gel and electro-transferred to PVDF membrane (Millipore, Billerica, MA). The membrane was blocked with 5% non-fat milk in PBS at 4 °C overnight. The PVDF membrane was then incubated with rabbit anti-mouse-IL-15 polyclonal Ab followed by secondary Abs goat- anti-rabbit IgG (ABcam, Cambridge, MA) conjugated with HRP, and developed by ECL-advanced western detection kit (Cell Signaling Technology, Danvers, MA).

### EAE model

Female mice (7–8 weeks old) received an HGT injection 3 days before EAE induction and were divided into 3 groups as follows: mice received HGT injection of plasmid DNA encoding IL-15 gene; mice received HGT injection of plasmid DNA encoding IL-15ΔE6 gene; mice received HGT injection of minicircle plasmid. Subsequently, the mice were injected subcutaneously with 300 μg MOG (Sangon Biotech, Shanghai, China) in complete Freund’s adjuvant (Sigma-Aldrich, St. Louis, MO) mixed with 4 mg/ml *Mycobacterium tuberculosis* (BD Difco, San Jose, CA). 500 ng pertussis toxin (List Lab, Campbell, CA) was injected intraperitoneally on days 0 and 2. Clinical signs were assessed daily as follows: 0, no clinical signs; 0.5, partially limp tail; 1, paralyzed tail; 2, mild hind-limb paresis; 3, severe hind-limb paresis; 4, hind-limb paralysis; 5, hind limbs paralyzed and partial forelimb weakness. The clinical scores were evaluated and recorded in a double blinded fashion.

### Histology and immunohistochemistry

Brain and spinal cord tissues fixed in 10% phosphate-buffered formalin (pH 7.4) were dehydrated in 100% ethanol and stained with hematoxylin and eosin to identify areas of demyelination and the number of parenchymal inflammatory lesions. To determine the number of immune cells per section, the number of immune cells in three randomly chosen lesions was counted using ImageJ software. The resulting mean value of immune cells/mm2 was then multiplied by the total lesion area for that section to yield the total number of immune cells/section.

To detect the expression of IL-15 and IL-15 isoform in the mice after HGT treatment, liver tissues fixed in 10% phosphate-buffered formalin (pH 7.4) were dehydrated in 100% ethanol and embedded in paraffin wax at 58 °C. The paraffin sections were stained with a polyclonal rabbit Ab to murine IL-15 (Abcam, Boston, U.K.). The IL-15 Ab was diluted in PBS (pH 7.4) and applied at concentrations of 1:1000 at 37 °C for 30 min. Endogenous peroxidase activity was blocked with 3% H_2_O_2_ and methanol. Avidin and biotin pretreatment was used to prevent endogenous staining. The secondary Ab was biotinylated goat anti-rabbit IgG used at 1:2000 dilution (Abcam, Boston, U.K.). For the analysis of macrophage infiltration in CNS, brain and spinal cord tissues fixed in 10% phosphate-buffered formalin (pH 7.4) were dehydrated in 100% ethanol and embedded in paraffin wax at 58 °C. The paraffin sections were stained with a polyclonal rabbit Ab to murine F4/80 (Santa Cruz Biotechnology, Taxes) at concentrations of 1:500 followed by secondary Ab staining which was goat anti-rabbit IgG used at 1:2000 dilution. Color development was performed with the amino ethylcarbazole detection kit from Ventana Medical Systems (Beijing Biosynthesis Biotechnology, Beijing, China).

### Statistical analysis

One-way ANOVA was used to determine statistically significant differences among more than two experimental groups. Unpaired Student t tests were used to determine statistically significant differences between two experimental groups. Data were analyzed using GraphPad Prism 5 software for Windows (GraphPad Software, San Diego, CA). *P* value < 0.05 was considered statistically significant (*), less than 0.01 or 0.001 was shown as ** or ***, respectively.

## Results

### Exon-6 lacking IL-15 isoform can be generated by alternative splicing in activated B cells and macrophages

**Figure 1:**
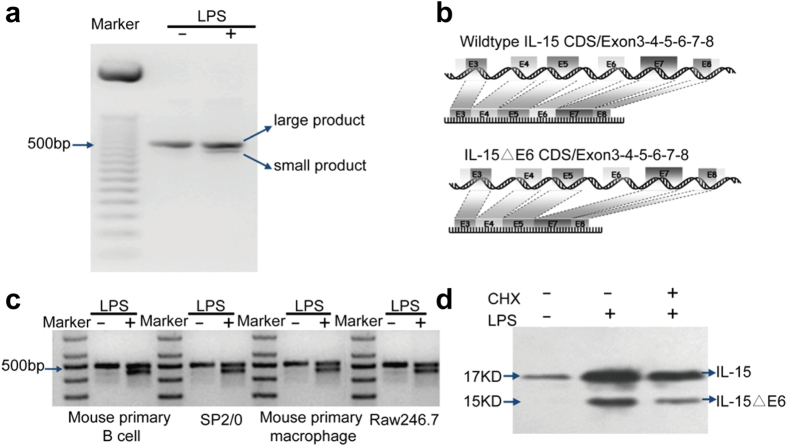
Murine exon-6-lacking-IL-15 isoform was generated by alternative splicing in activated immune cells. (**a**) Splenocytes were cultured with or without LPS (10 μg/ml) for 24 h. Total RNA extracted from the splenocytes was reverse transcribed into cDNA and amplified by PCR using a specific primer for coding sequences of murine IL-15 cDNA. (**b**) Coding sequence comparison between IL-15ΔE6 isoform and IL-15. Boxes represent exons and the dashed lines indicate the consequence of alternative splicing. (**c**) Primary B cells, macrophages, SP2/0 cell line and Raw 246.7 cell line were cultured with or without LPS (10 μg/ml) for 24 h. Total RNA was extracted from these cells and reverse transcribed into cDNA and amplified by PCR. (**d**) Splenocytes were cultured with or without LPS (10 μg/ml) for 24 h. During LPS stimulation, CHX (1 μg/ml) was added for the last 12 h of culture. The cellular lysates were blotted with rabbit anti-mouse-IL-15 polyclonal Ab followed by goat- anti-rabbit IgG Ab. The data shown represent at least three independent experiments.

It was reported that the level of IL-15 transcription was increased in the immune cells after stimulation with LPS^24^,^48^. Interestingly, we observed a smaller product of IL-15 from RT-PCR reaction using specific primers for IL-15 and the cDNA from splenocytes stimulated with LPS for 24 h (Fig. 1a). This smaller product was not present in the unstimulated splenocytes. The smaller PCR products were sequenced and proved to be the exon-6-missing IL-15 isoform (Fig. 1b).

As LPS mainly activates B cells and macrophages, the activation-induced production of IL-15ΔE6 could be from B cells and macrophages. In order to confirm that, we stimulated mouse primary B cells, macrophages, mouse myeloma cell line SP2/0 and mouse monocyte-macrophage leukemia cell line RAW246.7 with LPS (Fig. 1c). The RT-PCR results indicated that the expression of IL-15ΔE6 could be observed in all these cells after LPS stimulation. Primary T and NK cells served as negative control in these experiments and showed no IL-15 expression (Supplemental Fig. 1). Macrophages from TLR4 deficient mice failed to express IL-15ΔE6 upon LPS stimulation, suggesting that this isoform expression was indeed activation-induced (Supplemental Fig. 1). To further determine whether this IL-15 isoform was generated by an alternative splicing and not by enzymatic process of the protein, we treated LPS-stimulated splenocytes with cycloheximide (CHX), a drug that could inhibit protein translation (Fig. 1d). After 12 h of treatment, the expression level of IL-15ΔE6 was significantly reduced together with IL-15 in the LPS-stimulated splenocytes, maintaining a similar ratio as in the control cells, suggesting that the protein synthesis was required for the generation of IL-15ΔE6. These results demonstrated that IL-15ΔE6 was generated by alternative splicing in activated B cells and macrophages.

### IL-15ΔE6 suppresses IL-15-mediated T cell proliferation *in vitro*

**Figure 2:**
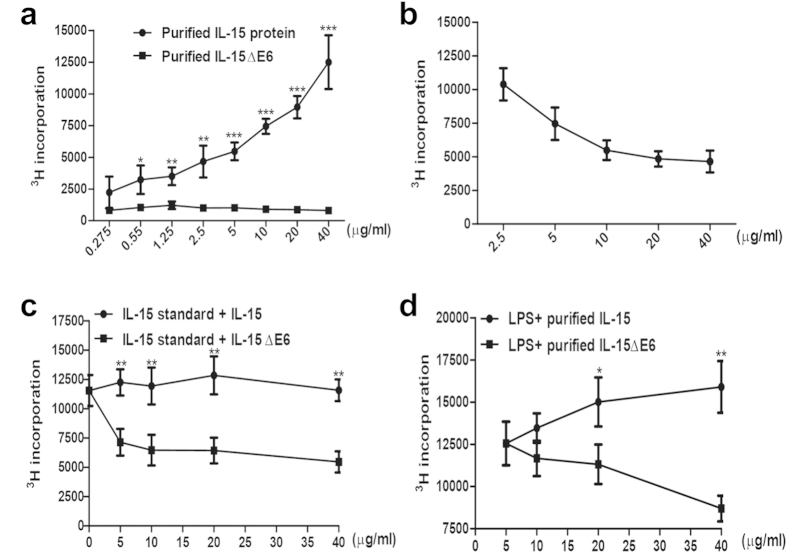
The effect of IL-15ΔE6 on IL-15-mediated CTLL-2 cell proliferation. (**a**) Titrated concentrations of purified IL-15 or IL-15ΔE6 were added to the CTLL-2 proliferation assay. (**b**) Titrated concentrations of purified IL-15ΔE6 protein was added to the CTLL-2 cells culture with 40 μg/ml purified IL-15. (**c**) Titrated doses of purified IL-15 or IL-15ΔE6 protein was added to CTLL-2 cell culture with 2.5 ug/ml commercially bought IL-15 standard. (**d**) The splenocytes were stimulated with 1.25 ug/ml LPS in the presence of increased concentrations of purified IL-15 or IL-15ΔE6 protein. The data represent three independent experiments and values are presented as means ± SD. *p < 0.05, **p < 0.01, ***p < 0.001.

In order to determine the biological function of IL-15ΔE6, both IL-15ΔE6 and IL-15 were expressed in *E. coli* and purified using a Ni-NTA agarose column. CTLL-2 is an IL-2 dependent T cell line which can also respond to IL-15. We cultured the CTLL-2 cells in the presence of IL-15ΔE6 or IL-15 (Fig. 2a). The recombinant IL-15 protein promoted the growth of CTLL-2 cells in a dose-dependent manner while recombinant IL-15ΔE6 protein couldn’t induce CTLL-2 cell proliferation. To further assess whether IL-15ΔE6 had an antagonistic effect on IL-15-mediated CTLL-2 cell proliferation, the recombinant IL-15ΔE6 protein were added to the CTLL-2 cells cultured with recombinant IL-15 (Fig. 2b). Addition of IL-15ΔE6 significantly down-regulated IL-15-mediated CTLL-2 proliferation in a dose-dependent manner.

To further confirm the antagonistic effect of IL-15ΔE6, commercially bought IL-15 standard was used in CTLL-2 culture and recombinant IL-15 or IL-15ΔE6 protein were added to the culture (Fig. 2c). As expected, IL-15 protein slightly promoted CTLL-2 proliferation. However, IL-15ΔE6 significantly reduced the proliferation of CTLL-2 cells supported by IL-15 standard. Since IL-15ΔE6 was identified in the LPS stimulated splenocytes, we also tested whether IL-15ΔE6 could exert inhibitory effect on splenocyte proliferation stimulated with LPS (Fig. 2d). LPS was used to stimulate murine splenocytes together with recombinant IL-15 or IL-15ΔE6. We found that IL-15ΔE6 effectively suppressed LPS-induced splenocyte proliferation in a dose-dependent manner. These results demonstrated *in vitro* that IL-15ΔE6 induced by stimulation could antagonize the function of IL-15 in promoting T cell proliferation.

### IL-15ΔE6 can bind to IL-15 Rα and compete with IL-15

**Figure 3:**
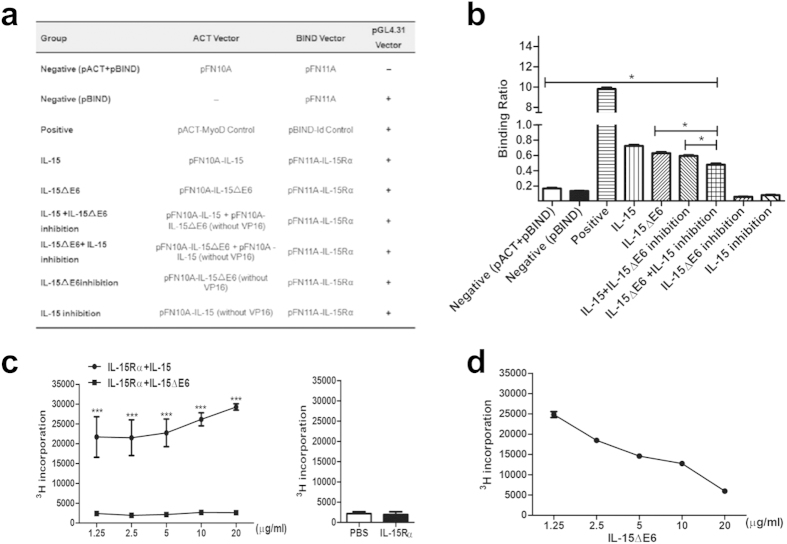
IL-15ΔE6 could interact with IL-15Rα and interfere with the binding of IL-15 to IL-15Rα. (**a**) Experimental groups for testing the interactions between IL-15 to IL-15Rα, as well as IL-15ΔE6 and IL-15Rα. (**b**) Summary data of binding activities in different groups. (**c**) CTLL-2 proliferation assays were performed in the presence of the mixture of IL-15Rα/IL-15 or IL-15Rα/IL-15ΔE6. (**d**) Titrated amounts of IL-15ΔE6 were added in the CTLL-2 proliferation assay with the mixture of IL-15Rα/IL-15. The data shown represent three independent experiments and values are presented as means ± SD. *p < 0.05, ***p < 0.001.

To explore the possible mechanism of the antagonistic role of IL-15ΔE6, we cloned the coding regions of IL-15, IL-15ΔE6 and IL-15Rα into the CheckMateTM/Flexi mammalian two-hybrid system (Fig. 3a). This system allows for protein expression and post-translational modifications and provides means to confirm interactions between two proteins (Cho *et al*.; Mahlknecht *et al*.; Wessels *et al*.). The pACT vector containing a herpes simplex virus VP-16 transcriptional activation domain was served as acceptors of IL-15 and IL-15ΔE6 protein-coding regions. The IL-15Rα coding region was inserted into the pBIND vector. The pGL4.31 vector containing five GAL4 binding sites was used as a reporter for interactions between proteins. To validate whether the binding between IL-15ΔE6 and IL15Rα could impact the binding efficacy between IL-15 and IL-15Rα, we also transferred the coding regions of IL-15 and IL-15ΔE6 into the pACT vector without VP-16 coding region respectively. We then transfected these purified vector constructs into CHO cell line. The control and experimental groups were set up according to Fig. 3a. The supernatant of cell lysis was collected 48 hours after transfection and read after adding the substrate of firefly luciferase and Renilla luciferase respectively. The result showed that IL-15ΔE6 could interact with IL-15Rα (Fig. 3b). It slightly inhibited the binding of IL-15 to IL-15Rα, but the difference did not reach statistical significance (The IL-15 group compared with the IL-15 plus IL-15ΔE6 inhibition group). Also in the presence of IL-15, the binding efficacy between IL-15ΔE6 and IL-15Rα was down-regulated (The IL-15ΔE6 group compared with the IL-15ΔE6 plus IL-15 inhibition group, *p* < 0.05). The extent of inhibition with IL-15 was a bit more than the inhibition with IL-15ΔE6, suggesting the affinity between IL-15ΔE6 and IL-15Rα might be slightly lower than that between IL-15 and IL-15Rα.

To further demonstrate the biological function of IL-15ΔE6 on the formation of IL-15/IL-15Rα superagonist, we performed proliferation assays with the CTLL-2 in the presence of IL-15/IL-15Rα superagonist or IL-15ΔE6/IL-15Rα mixture (Fig. 3c). The IL-15/IL-15Rα superagonist significantly promoted the proliferation of CTLL-2 cells. However, the mixture of IL-15Rα and IL-15ΔE6 did not have any effect on the proliferation. Adding increasing amounts of IL-15ΔE6 to the mixture of IL-15Rα and IL-15 dose-dependently reduced the proliferation of CTLL-2 cells (Fig. 3d), suggesting that IL-15ΔE6 could compete for the binding of IL-15Rα and interfere with the generation of the IL-15Rα/IL-15 superagonist.

### IL-15ΔE6 expression significantly reduces the severity of EAE

**Figure 4:**
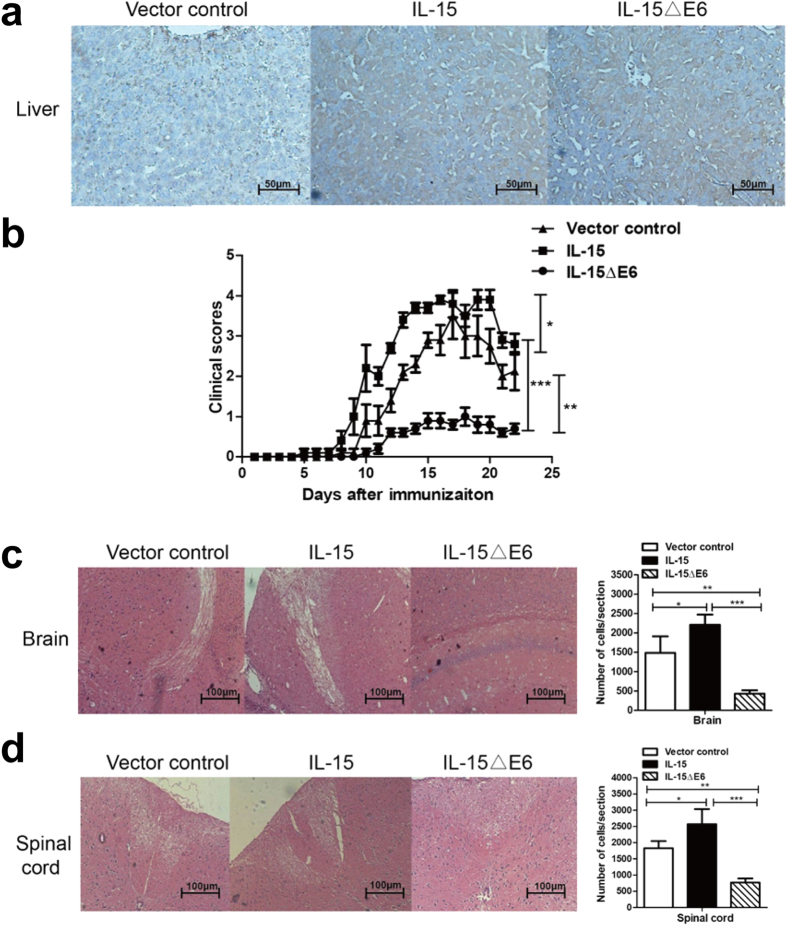
Mice expressing IL-15ΔE6 developed less severe EAE compared with control group and IL-15 group. (**a**) HGT method mediated high levels of IL-15 expression in the EAE mice. The liver sections of the mice received HGT were stained with polyclonal rabbit Ab to murine IL-15 and goat anti-rabbit IgG. Original magnification × 200. (**b**) EAE was induced in mice three days after HGT of IL-15-MC, IL-15ΔE6-MC vector and MC vehicle plasmid. The clinical scores were evaluated daily in a double blinded fashion. The graph showed the daily average clinical scores (mean ± SD). (**c**,**d**) The HE staining of spinal cords and brains from IL-15, IL-15ΔE6 and control mice with EAE. Original magnification × 100. The numbers of infiltrating immune cells were quantified and analyzed. The data shown are the representative of three experiments, each with 5–6 mice per group. *p < 0.05, **p < 0.01, ***p < 0.001.

In order to dissect the *in vivo* function of IL-15ΔE6, we overexpressed IL-15ΔE6 and IL-15 by hydrodynamic gene transfer (HGT) method in a murine EAE model. The IL-15 and IL-15ΔE6 expressions were quite sustainable. Liver tissues from mice that received HGT injection of IL-15 and IL-15ΔE6 plasmids showed a significantly higher and comparable levels of IL-15 detected by antibody that could recognize the common regions of IL-15 and IL-15ΔE6 (Fig. 4a).

The clinical scores of the control mice reached the peak (3.5–4) around day 14 post immunization (Fig. 4b). Mice received IL-15 HGT had earlier onset of the disease and increased severity compared with the control mice (*p* < 0.05). In marked contrast to IL-15 and the control group, over-expression of IL-15ΔE6 significantly reduced the severity of EAE as mice showed lower clinical scores and better movement compared to the control group (*p* < 0.01) and the IL-15 group (p < 0.001). None of the mice overexpressing IL-15ΔE6 had severe EAE symptoms (defined as a clinical score ≥ 3), only mild weakness of hind legs and tail atony was observed. As the injuries of myelin sheath and the inflammation in the CNS are the primary causes of EAE, we first validated whether the reduced EAE symptoms by IL-15ΔE6 were associated with lessened damage in CNS. As expected and consistent with the clinical score results, the HE staining of spinal cord and brain showed that the inflammatory lesions were fewer in the IL-15ΔE6 group compared with IL-15 group and the control group (Fig. 4c,d). IL-15 significantly increased the number of infiltrating immune cells in the CNS, while IL-15ΔE6 expression dramatically reduced it. These results demonstrated that IL-15 could aggravate EAE while IL-15ΔE6 reduced the severity of EAE, possibly by antagonizing the effect of IL-15.

### IL-15ΔE6 expression decreases the infiltration of macrophages in the CNS and down-regulates the percentages of inflammatory T cell subsets

**Figure 5:**
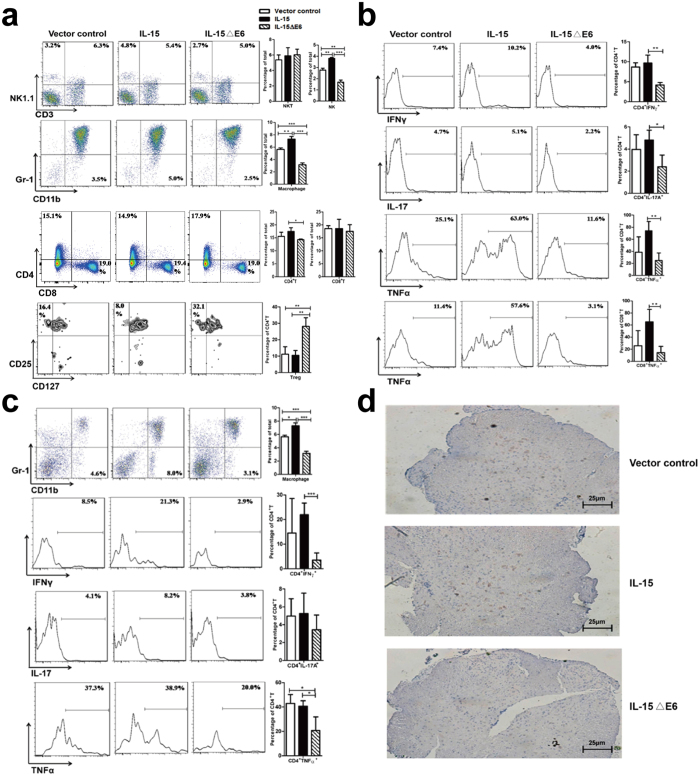
IL-15ΔE6 reduced the infiltration of macrophages into CNS and the percentages of inflammatory T cell subsets. (**a**) Flow cytometry analysis of the percentages of NK cells, NKT cells, CD4^+^ T cells, CD8^+^ T cells, macrophages, and Tregs, from the spleens of mice expressing IL-15, IL-15ΔE6 or control plasmid. (**b**) Percentages of CD4^+^IFN-γ^+^ T cells, CD4^+^IL-17^+^ T cells, CD4^+^TNF-α^+^ T cells and CD8^+^TNF-α^+^ T cells from the spleens of mice expressing IL-15, IL-15ΔE6 or control plasmid. (**c**) Percentages of macrophages, CD4^+^IFN-γ^+^ T cells, CD4^+^IL-17^+^ T cells and CD4^+^TNF-α^+^ T cells from the spinal cord of mice expressing IL-15, IL-15ΔE6 or control plasmid. (**d**) Spinal cord tissues from EAE mice that received HGT IL-15, IL-15ΔE6 or control plasmid were stained with polyclonal rabbit Ab to murine F4/80 and goat anti rabbit IgG. Original magnification × 400. The data shown represent three independent experiments (5–6 animals/group) and values are presented as means ± SD. *p < 0.05, **p < 0.01, ***p < 0.001.

Previous research had proven that many immune cell subsets, such as Th1, Th17 and macrophages participated in the course of EAE^49^,^50^ and regulatory T cells inhibited the disease progression^51^,^52^,^53^. To assess whether the reduced disease severity by IL-15ΔE6 expression was associated with the changes in the immune cell subsets, lymphocytes were acquired from spleen and spinal cord of EAE mice and stained with antibodies specific for different cell surface and intracellular markers (Fig. 5). By flow cytometry analysis, we found that the proportions of NK cells and macrophages were significantly reduced in the spleens of mice expressing IL-15ΔE6 compared with control group and IL-15 group (Fig. 5a). Percent of CD4^+^ T cells was slightly reduced in the IL-15ΔE6 group compared with the IL-15 group, while CD8^+^ T cells and NKT cells remained unchanged among three groups. Meanwhile, the percentages of regulatory T cells were increased by IL-15ΔE6 expression (*p* < 0.05).

Th1 and Th17 cells are commonly considered to be the key cell subsets in the occurrence and development of EAE. We further assessed the changes of Th1 and Th17 cells by intracellular staining (Fig. 5b,c). The percentages of CD4^+^IFN-γ^+^ T and CD4^+^IL-17^+^ T cells were significantly down-regulated in the spleens of mice expressing IL-15ΔE6 compared with the IL-15 group (p < 0.01, *p* < 0.05) (Fig. 5b). The percentages of CD4^+^TNF-α^+^ and CD8^+^TNF-α^+^ T cells were also decreased in the IL-15ΔE6-expressing mice (*p* < 0.01). Similar results were obtained in the CNS. We found a significant increase of macrophage infiltration in the IL-15 group (*p* < 0.05) while the macrophage infiltration was reduced in the IL-15ΔE6 group compared with the IL-15 and the control group in the spinal cord (*p* < 0.001, Fig. 5c). These results were also confirmed by immunohistochemistry analysis of macrophage infiltration in the CNS (Fig. 5d). The percentages of both CD4^+^IFN-γ^+^ and CD4^+^TNF-α^+^ T cells were significantly decreased in the 15ΔE6-expressing mice (p < 0.001, *p* < 0.05) (Fig. 5c). Taken together, our results demonstrated that IL-15ΔE6 expression decreased the infiltration of macrophages in the CNS and down-regulated the percentages of both CD4^+^IFN-γ^+^ and CD4^+^TNF-α^+^ T subsets in both spleen and CNS.

## Discussion

IL-15 expression is tightly regulated at multiple levels due to its important immunomodulatory function^18^,^19^. The interferon response factor element (IRF-E) and nuclear factor NF-κB are critical for regulation of IL-15 mRNA expression^20^,^54^,^55^. In terms of translational control, multiple upstream AUGs in the 5′-UTR, regulatory sequence in the C terminus of IL-15 mRNA, and long signal peptides tightly regulate translation and secretion of IL-15^21^,^56^. In addition, IL-15 expression can be controlled by alternative splicing which has been implicated in various aspects of cytokine production and signaling^21^,^57^. In this way, cytokine diversity is generated without the requirement for the expression of a new gene^58^,^59^. In the current study, we identified an IL-15 isoform lacking exon-6, IL-15ΔE6, generated by alternative splicing events of activated immune cells, including macrophages and B cells. *In vitro* data confirm that IL-15ΔE6 can antagonize IL-15-mediated T cell proliferation. Receptor binding assay reveals that IL-15ΔE6 can bind to IL-15Rα and interfere with binding between IL-15 and IL-15Rα. Over-expression of IL-15ΔE6, in a murine EAE model, dramatically ameliorated EAE symptoms and reduced the percentages of inflammatory T cells in the spleen and spinal cord of the EAE mice, as well as inhibited infiltration of macrophages to the CNS. Our data show that IL-15ΔE6 could be induced during immune activation and function as a negative feedback mechanism to dampen IL-15-mediated inflammatory events.

Five variants of IL-15 have been identified so far, including all combinations with an exon 2 and exon-6 deletion, a portion of exon 7 deletion and an alternative use of exon 5 or 5′^20^,^23^,^24^,^25^. All of these variants encode the same mature IL-15 protein except for variants lacking exon 6 and a portion of exon 7. Although the exon 6-missing IL-15 isoform was first identified in mouse intestinal epithelium^25^, we reported, for the first time, that it can be generated by alternative splicing events in the activated immune cells. For many known cytokines, including IL-2 and IL-4, alternatively spliced isoforms are natural antagonists with competitive inhibitory effects on cytokine function^60^,^61^,^62^. Of note a report indicated that when macrophages were stimulated with LPS, an IL-15 isoform utilizing alternative exon 5 and exon 2 deleted-isoform were obtained^48^. This discrepancy between their results and ours may be due to the short period of stimulation in their study (6 h). We stimulated splenocytes, macrophages and B cells with LPS for 24 h to in order to observe the generation of IL-15ΔE6. This negative feedback mechanism mediated by IL-15ΔE6 may occur during late-stage of activation.

IL-15 is critical for autoimmune and inflammatory disease and previous studies show that the increased expression of IL-15 is associated with rheumatoid arthritis^63^,^64^,^65^, sarcoidosis and inflammatory bowel disease^29^,^66^,^67^. IL-15 also contributes to MS^38^,^39^,^40^. IL-15 up-regulation is observed in serum and cerebrospinal fluid of MS patients^41^,^42^ and mice of experimental autoimmune encephalomyelitis (EAE)^2^. IL-15 contributes to the tissue damages caused by CD8^+^ T cells and Th17 cells in MS patients and EAE models^45^,^46^. In addition, IL-15 is found to actively modulate microglial reactivity, co-activating the MAPK and NF-κB pathways, controlling additional cytokine and chemokine release^38^. Therefore, IL-15 may contribute to the disease progression of MS and EAE. Consistent with these previous reports, our results showed that mice over-expressing IL-15 had accelerated and more severe EAE (Fig. 4). Furthermore, the IL-15E6 isoform, which can negatively regulate the function of IL-15, alleviated EAE symptoms. However, IL-15 knockout mice exhibits aggravated EAE symptoms^2^,^44^,^68^. Because IL-15 is involved in NK and NKT cell development and in homeostatic maintenance of the T cells, the knockout mice data may not represent effector functions of IL-15 during EAE development. Rather, they may indicate that IL-15 is important for the development of a cell subset that could be protective against EAE. Moreover, IL-15 treatment did not significantly affect disease severity in both WT and knockout mice in the same report^2^. Differences in our and other’s results in terms of IL-15 treatment in EAE may be due to the level of IL-15 proteins. The HGT method using minicircle vector enables more efficient and sustained expression over a period of weeks compared to injection of recombinant proteins that only works for a few days^69^,^70^. Therefore, high IL-15 expression may result in the pathological role of IL-15 we observed during EAE development.

Although IL-15 mainly function on CD8^+^ T and NK cells, it could also influence the differentiation and maintenance of the CD4^+^ T cell subsets by modulating the specific signaling pathways together with IL-15Rα^71^,^72^. We found that IL-15ΔE6 could interact with IL-15Rα and partially inhibit binding of IL-15 to IL-15Rα which can be presented *in trans* to the IL-2Rβγc complex and initiates a series of signaling events involving JAK/STAT, phosphoinositide 3-kinase (PI3K) and the mitogen-activated protein kinase (MAPK) signaling pathways^73^. The binding of IL-15ΔE6 to IL-15Rα may prevent its association with the IL-2Rβ/γc to activate down-stream signaling pathways. However, how IL-15ΔE6 might interact with IL-15Rα and affect the IL-15 signaling in inflammatory conditions requires further investigation.

In conclusion, we identified an IL-15 mRNA isoform lacking exon 6, IL-15ΔE6, in activated macrophages and B cells which acts as a natural antagonist for IL-15 and may hold promise as a novel therapeutic agent for IL-15-mediated inflammatory diseases.

## Additional Information

**How to cite this article**: Zhao, L. *et al*. An activation-induced IL-15 isoform is a natural antagonist for IL-15 function. *Sci. Rep.* **6**, 25822; doi: 10.1038/srep25822 (2016).

## Supplementary Material

Supplementary Information
