# Supplementary material for: An activation-induced IL-15 isoform is a natural antagonist for IL-15 function
Source: Sci Rep. 2016 May 11;6:25822. doi: 10.1038/srep25822 (PMC4863161; doi:10.1038/srep25822)
Supplement: Supplementary Information [file srep25822-s1.pdf]

An activation-induced IL-15 isoform is a natural antagonist for IL-15 function

Lei Zhao, Bo Hu, Yinsheng Zhang, Yuan Song, Dandan Lin, Yonghao Liu, Yu Mei, Dedy Sandikin, Weiping Sun, Min Zhuang and Haiyan Liu

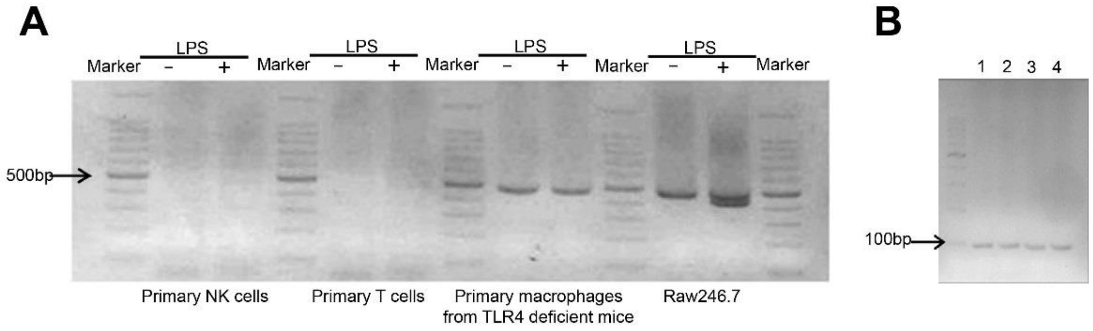

Supplemental Figure Legend:

Supplemental Figure 1. Control experiments for Figure 1C. (A) Experiments were performed in parallel with the experiments in figure 1C using primary T cells, NK cells from C57BL/6 mice or macrophages from TLR4 deficient mice. IL-15 products were generated by RT-PCR. (B) Actin controls for 1. NK cells, 2. T cells, 3. Macrophages, and 4. RAW246.7 cells.
